# Supplementary material for: Deletion of the chd7 Hinders Oligodendrocyte Progenitor Cell Development and Myelination in Zebrafish
Source: Int J Mol Sci. 2023 Aug 31;24(17):13535. doi: 10.3390/ijms241713535 (PMC10488005; doi:10.3390/ijms241713535)
Supplement: Supplementary file 1 [file ijms-24-13535-s001.zip › ijms-2569262-Supplementary.pdf]

Supplementary Figures

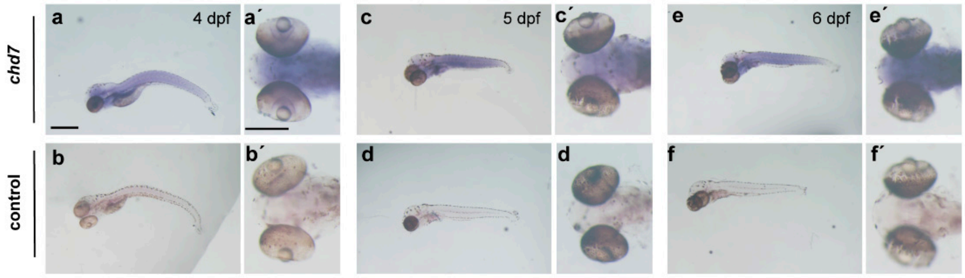

Fig. S1. The temporal and spatial expression pattern of *chd7* at 4-6 dpf.

a-f) Zebrafish at 4-6 dpf using WISH displayed high expression of *chd7* in the brain, eyes and spinal cord (lateral view). **a’-f’)** Dorsal view of the brain. Scale bar in a-f, 10 µm; Scale bar in a’ -f’, 5 µm.

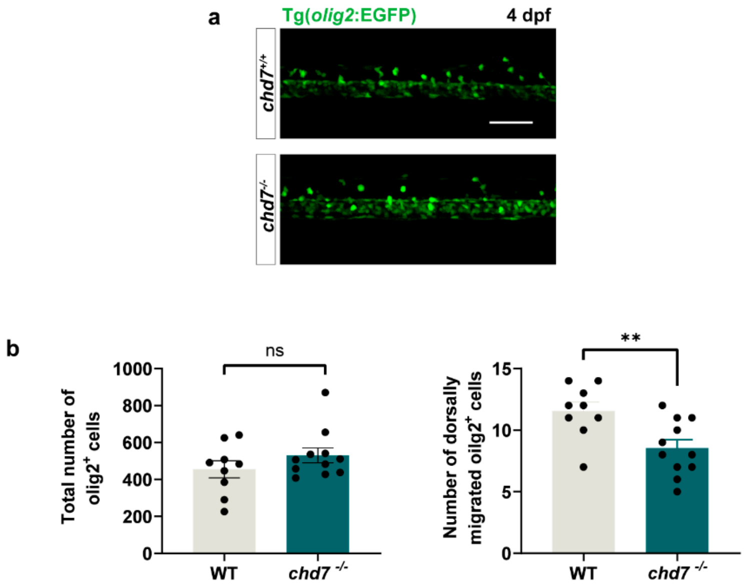

Fig. S2. Total number of OPCs were not significantly changed after *chd7* deletion.

a) Representative image of total and dorsally migrated olig2^{+} cells at 4 dpf between *chd7*$^{+/+}$ and *chd7*$^{-/-}$ group. Scale bar: 10 μm. **b)** The number of total olig2^{+} cells at 4 dpf did not occur significantly difference between the two groups. **c)** The number of dorsally migrated olig2^{+} cells at 4 dpf decreased in *chd7*$^{-/-}$ group. **$p = 0.0075$, unpaired Student's two-tailed *t*-test.
